# Gene Therapy in Retinal Dystrophies

**DOI:** 10.3390/ijms20225722

**Published:** 2019-11-14

**Authors:** Lucia Ziccardi, Viviana Cordeddu, Lucia Gaddini, Andrea Matteucci, Mariacristina Parravano, Fiorella Malchiodi-Albedi, Monica Varano

**Affiliations:** 1 Fondazione Bietti—IRCCS, Via Livenza 3, 00198 Rome, Italy; mariacristina.parravano@fondazionebietti.it (M.P.); monica.varano@fondazionebietti.it (M.V.); 2 National Center for Drug Research and Evaluation, Istituto Superiore di Sanità, Viale Regina Elena 299, 00161 Rome, Italy; lucia.gaddini@iss.it (L.G.); andrea.matteucci@iss.it (A.M.); fiorella.malchiodialbedi@iss.it (F.M.-A.)

**Keywords:** hereditary retinal disease, retinal gene augmentation, editing, optogenetics and splice modulation therapy, human iPSC-derived retina and retinal pigment epithelium, retinal pathology, pre- and clinical gene therapy, animal models for retinal dystrophy, retinal imaging

## Abstract

Inherited retinal dystrophies (IRDs) are a group of clinically and genetically heterogeneous degenerative disorders. To date, mutations have been associated with IRDs in over 270 disease genes, but molecular diagnosis still remains elusive in about a third of cases. The methodologic developments in genome sequencing techniques that we have witnessed in this last decade have represented a turning point not only in diagnosis and prognosis but, above all, in the identification of new therapeutic perspectives. The discovery of new disease genes and pathogenetic mechanisms underlying IRDs has laid the groundwork for gene therapy approaches. Several clinical trials are ongoing, and the recent approval of Luxturna, the first gene therapy product for Leber congenital amaurosis, marks the beginning of a new era. Due to its anatomical and functional characteristics, the retina is the organ of choice for gene therapy, although there are quite a few difficulties in the translational approaches from preclinical models to humans. In the first part of this review, an overview of the current knowledge on methodological issues and future perspectives of gene therapy applied to IRDs is discussed; in the second part, the state of the art of clinical trials on the gene therapy approach in IRDs is illustrated.

## 1. Introduction

The neuroretina is a light-sensitive membrane located in the back of the eye, with specialized sensory properties to enable the capture of light by photoreceptors, its conversion into electric signals through a phototransduction process, and further integration and processing of the electric impulses into an image at the central nervous system (CNS) level.

Due to its embryologic origin, the ultraspecialized anatomical and functional properties of the visual neurons, and the complex and refined biochemical processes of the visual cycle, the retina can be considered an extension of the CNS. Indeed, the retina is a window to the brain and a unique and accessible site for studying neurodegeneration phenomena [1].

**Figure 1:**
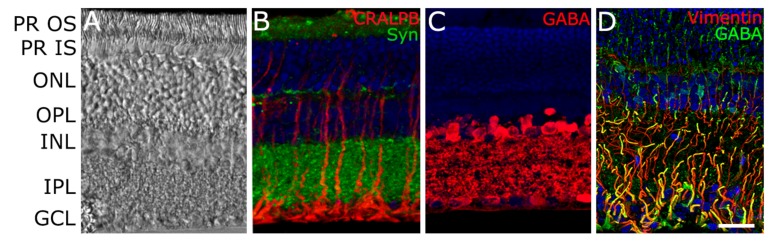
Retinal structure under light microscopy. The photographs highlight its histological complexity and evidence the connections between the different cell populations. (**A**) A section of rat retinal tissue, photographed using a differential interference contrast microscope, highlighting retinal layers. (**B**) Synaptophysin (Syn)-positive dots decorate the IPL, indicating the presence of synapses. In red, cellular retinaldehyde-binding protein (CRALBP)-positive Müller glia, spanning through all the retinal layers. (**C**) GABAergic neurons (GABA) in the inner layers of the retina. (**D**) A diagonal cross section reveals the rich connections between vimentin-positive Müller glia and GABAergic neuronal components. Nuclei are stained in blue with DAPI. PR OS = photoreceptors, outer segment; PR IS = photoreceptors, inner segment; GCL = ganglion cell layer; OPL = outer plexiform layer; ONL = outer nuclear layer; IPL = inner plexiform layer; INL = inner nuclear layer. Scale bar = 20 μm.

The retina has a well-organized architecture and is stratified in layers, where different cells are connected together (Figure 1). The single-layered retinal pigment epithelium (RPE) plays an essential role in supporting the metabolism of photoreceptors. These light-sensing cells which form the outer retina are categorized into cones and rods based on their ability to be more active in bright or dim light, respectively. At this level, phototransduction takes place and light is converted into an electric signal transmitted to the synaptic terminal of bipolar cells. These interneurons, enclosed in the inner retina, are the real connecting elements to the retinal ganglion cells (RGCs) whose fibers form the optic nerve. In the context of the neurosensory retina, other elements, such as amacrine cells, horizontal cells, and Müller cells, contribute to the retinal biochemical circuits and structure [2].

Due to its favorable anatomical and immunological characteristics, the eye has been at the forefront of translational gene therapy for inherited retinal dystrophies (IRDs), which are progressive degenerative diseases caused by a lack of normal proteins encoded by specific genes involved in retinal cellular structures, phototransduction, or the visual cycle [3].

Advantages of applying gene therapy to treat IRDs reside in (1) the accessibility of imaging and surgery with innovative and non-invasive techniques; (2) the tiny size of the retina, which requires small amounts of therapeutics to be administered; (3) the ocular immune privilege, due to the presence of the blood–retinal barrier [4], and the lack of lymphatic vessels, although the issue is still controversial [5], all of which make the eye a compartmentalized reservoir with privileged immunological defenses for good tolerance of gene therapy [6]; (4) the possibility of treating one eye and considering the untreated eye as an ideal control for the efficacy and safety of the treatment, with IRDs being bilateral; and, finally, (5) the availability of a wide range of animal models to test experimental therapies [7]. A main disadvantage is the post-mitotic nature of retinal tissues. First of all, vectors that are only able to penetrate cells during cell division have limited access. Furthermore, therapeutic success depends on the level of retinal degeneration at the time of treatment, since neuronal cells cannot be replaced once they degenerate.

At present, we have learned from the safety studies of ongoing clinical trials that exposure to the viral vectors of gene therapy can be well tolerated, especially when systemic oral steroids are administered as precaution [8].

In the delivery of agents for gene therapy, two main modes of administration have been considered: subretinal and intravitreal injections. Subretinal injections, which are more invasive, aim to reach the RPE and photoreceptor area more precisely. The subretinal bleb requires development of a transitory iatrogenic neuroretinal displacement very close to the fovea, which needs to be carefully controlled in order to prevent macular holes and retinal detachment. Intravitreal injections are easier to perform and less risky, and are considered better for targeting the inner retina, which is adjacent to the vitreous cavity. However, this method is more likely to solicit an immunological response because of the possibility of wider systemic spread of the agent [9].

## 2. Inherited Retinal Dystrophies (IRDs)

IRDs, known also as retinal degenerations or dystrophies, are a large group of inherited eye disorders resulting in irreversible visual loss and blindness. They develop due to mutations in one or more genes that lead to the death of the retinal photoreceptor cells. These specialized cells contain photopigments reactive to the light, made up of a protein (opsin) linked to a subform of vitamin A (retinal). The phototransduction begins at the level of photoreceptor outer segments, but to be effective, it requires the integrity of the RPE, where the regeneration of the photochemically active form of retinal (vitamin A)-11-cis retinal, phagocytosis of shed photoreceptors, and transport of nutrients and ions occur. Due to their extremely high levels of energy consumption, the photoreceptors receive high quantities of metabolites and oxygen through the choriocapillaris, a network of blood vessels of the choroid [1].

IRDs are neurodegenerative pathologies with a wide spectrum of presentations, even in the same affected family members, and are genetically heterogeneous. In addition, the rate of progression and severity of the disease show wide variety across different IRDs, as well as between different patients with the same retinal dystrophy. Mutations of about 270 different genes involved in eye development, photoreceptor survival, phototransduction mechanisms, retinoid cycle, retinal enzymatic function, or cell structure are responsible for these degenerative diseases (RetNet. Available at: https://sph.uth.edu/retnet/). The inheritance pattern can be autosomal dominant, recessive, *X*-linked or mitochondrial.

Disease onset may vary from birth to early childhood, adolescence, or adulthood. Even age-related degeneration may have some phenotypes resembling late-onset IRDs.

The common classifications for differentiating forms of retinal dystrophies are based on the primary involvement of specific cellular elements (rods, cones, or both, RPE, inner retina) or on the central or peripheral involvement of the retina, distinguishing maculopathies from retinopathies.

IRD patients are remarkably and progressively visually impaired and can be followed up by visual acuity measurements, visual field evaluations, electroretinographic recordings (ERG), structural imaging with autofluorescence, spectral-domain optical coherence tomography (OCT), and OCT angiography [10].

Although an accurate clinical diagnosis can be reached by these innovative and non-invasive tools, genetic testing is necessary to confirm a specific phenotype, and segregation analysis can address the inheritance pattern with greater precision. Some IRDs are monogenic or digenic, however in some cases the same gene can encode for different phenotypes. For example mutations of the *PROM1* gene have been shown to result in retinitis pigmentosa (RP) but also in Stargardt disease; of interest, in addition, is the broad spectrum of diseases seen in patients with *ABCA4* retinopathies, from Stargardt disease/fundus flavimaculatus, to cone–rod dystrophy to RP. This extreme genetic heterogeneity is the basis for about 30% of the detection failure of molecular diagnosis. In fact, although brilliant and unique clinically refined diagnoses can be made, genetic confirmation is still missing in one out of three cases of IRDs. To meet the important need for genetic diagnosis and to amplify the detection rate of the causative mutation, new and fascinating diagnostic avenues have been explored, starting from single-gene DNA sequencing (using the Sanger technique), moving to sequencing of panels of genes (by next-generation sequencing (NGS)) of the whole exome (by whole exome sequencing (WES)) up to investigating the entire genome (by whole genome sequencing (WGS)) [11,12]. The spread of such innovative technologies among genetic laboratories will certainly improve the diagnostic potential for IRDs.

Due to the heterogeneous presentation of IRDs, the congruence of clinical and molecular diagnosis is a necessary goal to characterize the phenotype exactly and to increase the chance of therapeutically beneficial strategies. The precise identification of genetic variants also gives the opportunity to inform the proband’s family members of the risk of recurrence of the disease, allowing pre-symptomatic diagnoses in younger family members to be made and helping in the recognition of related systemic symptoms when the disease is a syndromic disorder.

Overall, in the field of ophthalmic genetics, the main outcomes are (1) to introduce genetic tests into daily clinical practice so that they can be accessible and become a routine-based prescription; (2) to create genetic panels that, in conjunction with an exact clinical diagnosis, may result in informative and accurate diagnoses; (3) to increase the molecular test sensitivity for reaching higher mutation detection rate; and (4) to open up therapeutically personalized strategies for curing the disease.

A major challenge exists in identifying novel genes responsible for causing disease. With the possibility of investigating whole exomes or genomes, broader windows are opened for gene discovery. At present, molecular researchers, in close collaboration with bioinformatics, work to discover new disease-related genes, unveil noncoding mutations in known genes, and implement methodologies for detecting rearrangements and variants in distant promoter/enhancer regions, copy number variants (CNVs), and structural variations (SVs) in patients affected by IRDs. This research may also improve the ability to identify potentially pathogenic mutations and solve variants of uncertain significance (VUSs). It may also allow identification of phenotype-modulating genes through cohort studies of patients with mutations in the same gene but with different phenotype severities [12,13,14].

## 3. Gene Delivery Systems

In recent years, a plethora of viral and nonviral vectors have been evaluated for their transduction efficacy in the cells most affected by retinal degeneration, namely, RPE and photoreceptor cells.

The success of gene therapy depends on the vector choice (Table 1). Retinal dystrophy requires permanent correction, hence the need for transgene expression to last as long as possible and to have low immunogenicity, since the host’s immune response determines short-term transgenic expression [15,16].

Vectors that are not integrated into the host genome are preferable because the integration could cause adverse events following insertional mutagenesis. Other desirable features of a gene transfer vector include cellular specificity, where only target cells are transduced, with large or unlimited cloning capacity, lack of toxicity, and feasibility of being produced at high titers and levels of purity.

Recombinant virus vectors that are replication deficient, including adenovirus, lentivirus, and adeno-associated virus (AAV), have been widely used as vehicles to deliver the normal gene into diseased retinas and supplement the functional protein in targeted cells [17,18,19,20].

### 3.1. Viral Vectors

#### 3.1.1. Adenoviruses

Adenoviruses are the most frequently used group of gene therapy vectors due to their capacity to be successfully transduced into a large number of cell types, as well as to obtain high levels of transgene expression independent of the cell cycle stage. However, transduction of photoreceptors via adenoviral vectors is poor, except in the developmental phases of the cell type, such as in newborn mice or during the process of degeneration, as in adult *rds* mice models. This is likely due to the conformation and architecture of the normal adult mouse retina, which can act as a barrier to gene transfer mediated by viral vectors [21].

Adenoviral vectors seem to be more suitable for the treatment of RPE defects. Vollrath et al. demonstrated an improvement in photoreceptor function and number in areas injected with rAd that expressed the *MERTK* gene in the RCS rat up to 1 month after treatment [22].

Compared to other vectors, adenoviral vectors have the advantage of being able to carry very large inserts, up to 48 kb, but they have limits in terms of their safety and the lack of longevity of expression, due to the expression of some viral genes of the vector backbone in the transduced cells (Table 1). This triggers an immune response against the transduced cells, resulting in only transient transgenic expression and long-term toxicity [23,24]. This makes the adenoviral vectors unsuitable in pathologies in which long-lasting transgenic expression is needed. To make the adenoviral vectors safer and more efficient, some researchers have tried to incorporate fewer viral genes into the recombinant virion [25,26].

The first-generation vectors are deleted in the E1 region, making them replication defective, as well as in the E3 region, which enable these vectors to carry DNA inserts of up to 7.5 kb [26,27]. Newer second-generation adenoviruses have been engineered with additional deletions or mutations in the viral E2 and E4 regions, preventing transcriptional control of viral gene expression and viral genome replication, respectively [28,29]. Further improvement in the safety and efficacy of adenoviral vectors has come with the development of helper-dependent adenoviral vectors that are stripped of all viral coding sequences. They contain only the viral inverted terminal repeats and packaging sequences [30,31]. They retain the advantages of the first-generation adenoviral vectors in terms of high efficiency in in vivo transduction and transgene expression, and are able to mediate high-level, long-term transgene expression in the absence of chronic toxicity [32,33,34]. However, problems associated with these techniques, such as contaminating helper viruses, vector instability, and the emergence of replication-competent adenoviruses, have been reported [30,31,35].

#### 3.1.2. Lentiviruses (LVs)

LVs are RNA viruses of the retrovirus family possessing a reverse transcriptase through which they are able to integrate their retrotranscribed proviral DNA into the chromosomes of host cells. In IRDs, the retroviral variant of human immunodeficiency virus type 1 (HIV-1) or the equine infectious anemia virus (EIAV) have been tested. LVs have the ability to pass through the intact nuclear membranes and infect dividing cells [36], while retroviruses are able to infect dividing cells only when the nuclear membrane dissolves during cell division, making them unsuitable for use in post-mitotic tissues such as retina. LVs efficiently integrate their genome into that of the host cell, leading to stable expression (Table 1). This, however, increases the risk of insertional mutagenesis and has led to the development of non-integral vectors that have comparable transduction efficiencies and persist as episomal double-stranded DNA circles capable of transducing nondividing cells [37,38,39]. These vectors are rendered deficient in integrases by the introduction of a point mutation in the integrase gene. Efficient and sustained transgenic expression was demonstrated in vivo using non-integral lentiviral vectors in post-mitotic tissues, such as RPE, and at levels equivalent to those of their competent counterparts for integration [40]. LVs are modified to stop replication, so these vectors are not pathogenic after initial gene delivery, incorporating additional safety features that can facilitate their clinical application. LVs present a transgene capacity cargo of ~8–10 kb and, in terms of IRD, are capable of infecting RPE cells and, to a lesser extent, differentiated photoreceptors [41].

EIAVs have been used for *ABCA4*, as the size of its cDNA (6.8 kb) outplaces the cargo capacity of AAVs but not that of lentiviruses [18]. To assess this strategy in human subjects, a clinical trial (NCT01367444) has been ongoing for several years (Table 2), but no clear efficacy data have been reported. So far, the use of lentiviral vectors for gene therapy in the eye appears to be limited to genetic correction in RPE as this is the only type of cell transduced with sufficiently high efficiency for therapeutic purposes. Lentiviral vectors can be useful for the treatment of photoreceptor defects by mediating the delivery of secreted factors, such as neurotrophic or antiapoptotic factors, to RPE [38,42].

#### 3.1.3. Adeno-Associated Viruses (AAVs)

Among all the vectors that have been used in the eye for gene therapy, vectors based on AAVs represent the most promising approach for the treatment of many IRDs in terms of efficiency and stability of gene transfer. Several features render them suitable for retinal gene therapy, such as lack of pathogenicity, minimal immunogenicity, ability to transduce nondividing cells, and capacity to mediate sustained levels of therapeutic gene expression (Table 1). The main problems are represented by their limited packaging capacity, which precludes their use for the treatment of IRDs caused by mutations in genes whose coding sequence exceeds 5 kb. Therefore, in recent years, considerable effort has been made to identify strategies to increase the transfer capacity of AAV vectors [62].

Adeno-associated viruses are among the smallest viruses, with an uncoiled icosahedral capsid of about 22 nm. Since they require the presence of a helper virus for replication to occur, adeno-associated viruses are classified as dependoviruses that are naturally deficient in replication and nonpathogenic. An AAV is able to infect both dividing and nondividing cells and has a broad tropism that allows it to infect many cell types depending on the particular serotype [63,64]. To date, over 100 AAV serotypes of different animal species have been isolated [65,66,67,68]. The serotypes differ in the sequence of their capsid protein. The recombinant vectors of AAV (rAAV) used for gene therapy are mainly based on serotype 2 (AAV2); this was the first human serotype described and the best characterized AAV serotype. Since the AAV capsid protein is responsible for its tropism and, therefore, for its efficacy, a pseudotyped strategy was developed in which pseudotyped or hybrid AAV vectors encode a serotype rep, usually AAV2, and the cap gene of a different serotype.

Cell targeting strategies determine not only the types of cells with which the vector interacts, but also the efficiency of the gene transfer process, since cell penetration and intracellular trafficking depend directly or indirectly on the conformation of the capsid [69,70,71]. In addition to the pseudotype, other strategies for designing custom capsids have been employed to further expand the utility of AAV vectors. These include the generation of mosaic capsids (composed of a mixture of capsid subunits of different serotypes) or chimeric capsids (containing capsid proteins that have been modified by the domain or exchange of amino acids among serotypes). Recently, library selection and direct evolution have emerged as promising approaches to modulate AAV tropism [72,73,74,75,76,77,78,79].

In the context of ocular gene therapy, the pseudotype of the vectors remains the most important strategy to address gene delivery to specific cell types. They have been used to treat animal models of a wide range of retinal disorders from Leber congenital amaurosis (LCA) and RP to autoimmune uveitis and neovascular disorder [80,81,82,83,84,85,86,87,88,89,90,91,92]. They have also been used in clinical trials for the treatment of LCA [93].

### 3.2. Nonviral Gene Transfer

Although various viral vectors have demonstrated their potential in the treatment of IRDs, there is a persisting need for the improvement of delivery vectors. Therefore, research efforts have also been directed towards the development of nonviral delivery systems, such as nanoparticles (NPs), naked DNA, or liposomes [94], which are much cheaper and easier to produce in large amounts compared to viral vectors (Table 1).

Among the different methods, injecting or electroporating cells with naked plasmid DNA [95] are particularly promising considering their safety and biocompatibility. In addition, they have a low risk of inducing immune responses and can carry large coding sequences. Finally, more constructs can be simultaneously conveyed to the target tissue. The efficiency of gene transfer by the electroporation method is limited by the transiency of gene expression. In a study by Dezawa et al., intravitreous DNA plasmids were delivered into retinal ganglion cells in vivo [96], but the expression persisted only 21 days. In immature retina, the transfection was more efficient, and the reporter gene was expressed for up to 50 days after subretinal injection of a GFP expression vector in newborn mice [97]. The gene transfer efficiency of this in vivo electroporation method was sufficiently high to be useful in functional analyses using DNA-based RNA interference vectors (RNAi) and reporter constructs carrying specific retinal cell promoters or genes of interest.

Nanoparticles (NPs) can be used to provide plasmid DNA containing a functional copy of a gene in the retina [98]. Metal/polymer nanoparticle compositions have the ability to pass through the plasma membrane, escape endosomes, and transport the plasmid DNA to the nucleus. Lipidic NPs are stable and biocompatible, and do not cause immune responses after administration to the eye. However, some disadvantages have been reported, such as lower gene expression compared to that obtained with the same transgenes delivered using viral vectors [94]. DNA–gold NPs are a novel delivery system that are easy to generate and have high tolerability and low toxicity [99]. For the low clearance rate, this system has been further optimized for treating IRDs [100]. RPE cells responded differently to pristine gold NPs and DNA–gold NPs, probably because of changes in the membrane–NP interface and because of potential different alternative endocytic methods for internalization. Unsolved issues of this delivery system in the retina are the internalization routes, the exact nature of intracellular endosome trafficking, ways of silencing degradative pathways (such as the lysosomal pathway), and the potential to ensure constant expression of the therapeutic gene [99].

Another method of distributing DNA to cells is with liposomes. Lipofectin or cationic liposomes have been mainly used for in vitro gene transfer. Studies document that in the eye, liposome-mediated transfection can lead to the expression of the reporter gene in the retina, but the efficiency with respect to viral vectors is undoubtedly lower and the effects are transient [101]. Even if solutions are being sought to implement the transfection efficiency of liposome–cationic DNA complexes and the specificity of transduced cell types [102], it is unlikely that nonviral methods—while obviating the complications of viral vectors, such as immunological response, toxicity, and risk of oncogenesis—will ever reach the performance of the latter. Furthermore, liposomes present some specific limitations, such as the tendency to aggregate following administration as well as retinal toxicity [103]. For all these reasons, nonviral methods of DNA release do not seem promising for therapeutic use of IRDs, at least for the moment.

## 4. Gene Replacement

To date, the best results in the treatment of IRDs have been obtained using gene replacement strategies. The first indication of the therapeutic potential of gene replacement was obtained in a murine model of autosomal recessive RP, characterized by nonsense homozygous mutations in the *PRPH2* gene that encodes a membrane glycoprotein fundamental for the formation of disks in the outer segment of the photoreceptors. The subretinal injection of AAV2 carrying the *PRPH2* transgene allowed for the formation of disks and restoration of the structural integrity of photoreceptors [80] up to 42 weeks after the treatment (Table 1). However, long-term studies have noted that functional improvements are not maintained over time, and this is probably due to low levels of peripherin-2 protein and failure to block the loss of photoreceptors by apoptosis.

The gene replacement approach was also used in RCS rat, a well-characterized model of RP, with a mutation in the receptor tyrosine kinase, *MERTK*, which is exclusively expressed in the RPE [104]. In this condition, photoreceptors degenerate due to an excess of debris. The first experiments using recombinant adenoviruses to release the *MERTK* gene subretinally showed functional recovery of phagocytosis and a temporary delay in photoreceptor degeneration [22]. Other studies were conducted by delivering the *MERTK* gene with AAV [54] and LVs [105], showing a higher number of rescued photoreceptors and an improvement in electroretinographic function (ERG). However, ERG improvement lasted up to 4 months when LVs were used [105], compared to only for 9 weeks when using AAV2. [54]. Better results have been obtained in disorders in which genetic defects originate in the RPE (such as *RPE65-* and *MERTK-*mediated dystrophies) [54,105,106]. First of all, RPE is composed of a single cell layer, is easier to treat, and is more accessible. Furthermore, in several conditions, photoreceptor damage is secondary to RPE hereditary defects, and it is possible to restore the function and survival of photoreceptors by correcting the metabolic disorder in RPE.

Several studies have been conducted on Leber congenital amaurosis models. *Rpe65*^−/^^−^ mice were treated with AAV2/2 for replacement of the *RPE65* gene, which led to functional recovery of the photoreceptors without, however, slowing their degeneration [107]. A similar study, with some adjustment of the vector, showed a more substantial improvement in ERG, although the evaluation was limited to only 3 months and no information concerning the photoreceptor degeneration was reported [43]. These studies paved the way to the first approved gene therapy for IRDs (Luxturna, Table 2).

Mutations in the *ABCA4* gene cause Stargardt’s disease, a form of autosomal recessive juvenile macular degeneration. Although the *ABCA4* is expressed at the edge of the disc of photoreceptor external segments, the pathophysiology of the disease originates from the loss of RPE due to the accumulation of lipofuscin in these cells [108]. Until recently, *ABCA4* cDNA was considered too large to be packaged in AAV vectors, which are the only vectors capable of effectively transducing photoreceptors. However, the discovery that AAV5-based vectors can package large recombinant genomes up to 9 kb allowed studies of gene substitution in an *Abca4*^−/^^−^ mouse model of Stargardt’s disease. The subretinal injection of an AAV2/5 vector carrying *Abca4* led to a reduction in the lipofuscin content and to an improvement in the retinal morphology and function for up to 5 months [109].

Together, these studies suggest that gene replacement therapy directed at RPE represents a promising therapeutic strategy. Examples of successful gene therapy and cell replacement are discussed in the following.

## 5. Gene Silencing

Among the most common forms of autosomal dominant RP (adRP), approximately 30–40% of cases are caused by gain-of-function mutations in the *RHO* gene which encodes rhodopsin. This condition is characterized by a considerable heterogeneity of mutations, a characteristic shared by many dominant, inherited diseases. The mechanism underlying *RHO*-adRP is that the mutations produce altered proteins that interfere with the normal function of the wild-type protein, producing a toxic effect on cells. The therapeutic strategy is aimed at repairing or silencing the mutated gene (Table 1).

Two strategies for mRNA silencing have been proposed for the condition. The first includes the use of specific allele inhibitors that promote degradation of the mutated mRNA and allow the expression of the normal allele, sufficient to maintain the function of the photoreceptor cells [110]. The second strategy is a mutation-independent approach in which the transcripts of both alleles are suppressed and replaced by a nonsilenced wild-type form [111]. Both approaches can be mediated by a variety of antisense inhibitors such as ribozymes, antisense oligonucleotides, and small RNA inhibitors (siRNA).

### 5.1. Ribozymes

Ribozymes include all RNA molecules that have catalytic activity. Currently, hairpin and hammer ribozymes are the most commonly used to specifically inhibit gene expression due to their small size and their great specificity. These two groups of ribozymes require specific folding to promote site-specific cleavage of a target phosphodiester bond by a transesterification reaction. This particular secondary structure is induced by conformational changes in the presence of metal ions, such as magnesium, manganese, calcium, and cobalt ions, which act as cofactors [112,113]. Both mutation-dependent approaches are reported in the literature, such as the one used by Drenser and colleagues against the RHO p.P23H and p.S334Ter mutations, demonstrating the efficiency of an AIB-driven ribozyme in a P23H rat model [114,115], as well as mutation-independent approaches with ribozymes capable of cleaving rhodopsin mRNA in vitro [116,117,118].

Gorbatyuk et al. [118] firstly obtained a clear decrease in rhodopsin mRNA and a reduction of the amplitudes of the b wave after injection of the ribozyme by AAV transduction in a mouse model Rho^+/^^−^ at P6. Another more efficient ribozyme in a P23H rat model, expressing a transgenic copy of a mouse mutant allele and a copy of the wild-type rat allele, was tested in vivo by the same group. This ribozyme affects rhodopsin mRNA in dogs, mice, and humans, but not in rats. In this experiment, the non-targeted normal mouse copy simulated the effect of the replacement gene. They demonstrated a significant conservation (approximately 50%) of the amplitude of the a and b waves of the scotopic ERG in the treated eyes one month after injection [119]. However, the ERG response continued to decrease in the injected eyes at a speed parallel to non-injected eyes. This shows that ribozymes can only limit gene expression by in vivo targeted mRNA cleavage in the retina.

The recognition sequence of a ribozyme is highly represented in the genome, increasing the risk of off-target effects.

The use of allele-independent RNA inhibitors is more convenient since a single reagent can be used against different mutations in the same gene. For optimal therapy, this should be accompanied by simultaneous supply of the gene that codes for the wild-type protein [120]. To do this, it would be necessary to express both molecules in the same AAV vector. However, both sequences are too large to be packaged into a single AAV, and delivery will be a crucial point to consider for these strategies.

### 5.2. siRNA

Although the in vivo application of ribozymes has yielded promising results [119], an even more powerful and lasting approach can be obtained by RNA inhibition by small interfering RNA (siRNA) technology (Table 1).

RNA inhibition is a fundamental pathway by which a sequence-specific siRNA is able to target and cleave complementary mRNA. The process is triggered by the presence of long pieces of double-stranded RNA (dsRNA), which are cleaved into the fragments known as siRNA (21–23 nt long) by an RNase III enzyme called Dicer [121]. Alternatively, siRNA can be synthetically produced and then directly introduced into the cell, thus circumventing the Dicer mechanics [122,123].

The delivery of siRNAs to the posterior segment is an arduous task, since they must cross the vitreous body before reaching the retina. Usually, the most commonly used strategy is intravitreal rather than subretinal injection [124]. Since synthetic siRNAs can be degraded by endonucleases [125], multiple injections are generally required, with an increased risk of side effects, such as cataracts, retinal detachment, vitreous hemorrhage, and endophthalmitis [126].

To obtain a prolonged release of siRNA, some researchers have tried to encapsulate them in nonviral nanocarriers; however, the negative charge of the glycosaminoglycans interacts with the cationic transporters, limiting their diffusion and promoting their aggregation [103,127,128]. The use of polyethylene glycol to screen these nanospheres and avoid their aggregation [103] unfortunately also hampers their cellular uptake [129] and endosomal transport and release [130,131]. However, more promising results have been reported using TransIT–TKO transfection reagent as a carrier for intraocular siRNA delivery into the mouse retina after intravitreal injection [132].

Rhodopsin mRNA silencing by siRNA has been proposed to delay retinal degeneration in adRP using two different approaches. The first approach specifically silences the mutated alleles by blocking their transcription allowing, at the same time, the normal expression of wild-type alleles [133]. However, this approach was not sufficient to inhibit retinal degeneration in a transgenic rat model [134]. Furthermore, the considerable heterogeneity in rhodopsin gene mutations would require the development of individual therapies for each mutation, which would be an expensive and time-consuming process. An alternative strategy to overcome these limitations is RNA inhibition independent of mutation and gene substitution. In essence, the approach involves the suppression of mutant and wild-type alleles of the rhodopsin gene by siRNAs [119,135,136], and the concomitant supply of the rhodopsin gene, which is sequentially modified to evade suppression. Using this approach, O’Reilly and colleagues [120] demonstrated substantial inhibition of rhodopsin RNA and the expression of replacement genes in mice injected subretinally with adeno-associated viral vectors expressing both an siRNA and a replacement rhodopsin gene. Similarly, the intravitreal injection of this adeno-associated virus induced a short-term (10 days) beneficial effect in the outer nuclear layer of the mouse retina by simulating human RP linked to rhodopsin with the P23H mutation. More recently, long-term restoration of retinal structure and function was obtained in these P23H rhodopsin transgenic RP mice through mutant-independent rhodopsin siRNA in combination with rhodopsin RNA replacement [137].

Other genes associated with adRP, such as *RDS* and *RHO*, have likewise been a target for siRNA-based suppression coupled with gene replacement technology for the development of RP therapies [138,139].

These studies thus demonstrate that RNA inhibition, in combination with codon-modified gene substitution, can lead to photoreceptor salvage, as evidenced by structural and functional benefits following treatment in adRP animal models.

## 6. Antisense Oligonucleotides (AONs)

An alternative therapeutic approach able to correct aberrant splicing mechanisms is based on the use of AONs. AONs consist of DNA or RNA molecules which can be released as naked oligonucleotides or expressed by viral vectors such as AAV. Although not applicable to all mutations, AONs are ideal for blocking aberrant splicing events caused by activation of cryptic splicing sites, and thus restore normal splicing.

Compared to gene enhancement therapy, AON-based therapy has many advantages. Additionally, in this case, the eye immune privilege [6] confers the possibility of supplying AON molecules directly to the retina by intravitreal or subretinal injections, with minimal toxic immunological side effects [140].

Recently, two reports have shown the safety and effectiveness of retinal AON delivery. In one of the studies, the delivery of naked molecules aimed at rescuing CEP290 exons in the mouse retina resulted in a jump of the exon target. Furthermore, these AON molecules reached all the retinal layers and no adverse effects were detected [141]. In the other study, AONs were used to degrade the *RHO* mutant allele in autosomal dominant RP in rats [142].

In terms of future therapeutic intervention in humans, there are several pros and cons in the administration of AON based on AAV or in the delivery of AON as a naked molecule. Due to their small size, naked AONs may be able to penetrate and reach photoreceptor cells in the retina more easily after intravitreal injections as compared to subretinal delivery of the currently used AAVs. Furthermore, since naked AONs have limited stability (which varies from weeks to months), the potential negative side effects of AONs would probably also be transient. However, the use of naked AONs would require repeated and lifelong injections. By contrast, a single administration of effective AAV could give therapeutic benefits for years, potentially for life. Garanto et al. [143] described the therapeutic efficacy of AVV-mediated release of AON for the treatment of *CEP290*-related disease. In fibroblasts from patients with LCA, the aberrant *CEP290* junction was corrected, CEP290 protein levels were restored, and the ciliary phenotype was completely rescued after AON administration. In the mouse retinas of a humanized *CEP290* mouse model that carried the LCA mutation, the delivery of bare and AAV-mediated AON reduced the amount of aberrant CEP290 transcripts, demonstrating that both AON delivery methods provide an excellent strategy for the treatment of LCA associated with *CEP290* [143].

With this evidence, AON-based therapy could be an effective way to stop progression or even improve visual function in many visually impaired individuals worldwide.

## 7. Genome Editing: The CRISPR/Cas9 System

Due to the limited success of the gene replacement approach, which is very expensive, time-consuming, and not appropriate for autosomal dominant forms of IRDs, another interesting scenario involving innovative methodology is on the horizon: gene editing.

The CRISPR/Cas9 system is based on single-guide RNA (sgRNA), which transfers the nuclease Cas9 to target loci, where it generates double-stranded DNA breaks (DSB).

The specific detection of DNA point break by Cas nucleases depends on two sequences: the RNA guide, a sequence of 20 nt homologous to the target sequence, and the adjacent protospacer motif (PAM) of 3 nt. Any DNA sequence can be the target of the CRISPR/Cas system; the only requirement is that the target contains a PAM sequence. The most commonly used nuclease is Cas9 from *Streptomyces pyogenes* (SpCas9), which recognizes the PAM NGG sequence. Once the Cas nuclease is transferred into the cells, the double strand of the target sequence can be cut. DNA cutting can be repaired using two possible methods: nonhomologous end-joining (NHEJ) or homology-directed repair (HDR) [144,145].

NHEJ is an error-prone genome editing pathway that can introduce extra insertions and deletions (indels) in DSBs. These indels often introduce a reading frameshift in the sequence that can generate premature stop codons, forming a nonfunctional protein. This approach can be used to specifically inhibit the mutated allele, if the RNA guide only targets this allele, or both alleles (Table 1). If Cas9 recognizes both alleles, integration of the wild-type gene is required. NHEJ has been extensively used for gene deletion [146,147,148,149,150].

Conversely, HDR is a high-fidelity pathway for genome editing of germline or proliferating cells, although it is considered inefficient and relies on cell division [148,151,152,153]. During HDR, the cell uses the sister chromatids of the homologous chromosome as templates to repair the cut. Alternatively, a single-stranded donor oligonucleotides (ssODN) sequence can be provided, together with the Cas9 nuclease, as a template to repair in HDR mode. The limit of HDR is that it occurs much less frequently than NHEJ in the S and G2 phases of cell division. However, most cells in postnatal tissues in mammals, including neurons, gradually lose their proliferation capacity as they mature. Therefore, the Cas9-mediated HDR pathway has rarely been applied in postnatal tissues and cells. Photoreceptors and RPE, being postmitotic cells, lack the HDR repair mechanism. In a recent work, Cai et al. [154] aimed at designing a genome-based HDR-based editing technique to accurately correct the genetic mutation in *rd1* postnatal mouse retinas for the treatment of RP disease. They established a new Cas9/RecA system based on HDR which allowed for accurate correction of the Pde6b mutation and saved the photoreceptor from degenerating in rd1 mice in vivo. Compared to the NHEJ route, which has error-prone repair and poor high-fidelity changes, the HDR route has broader application in the treatment of diseases. Genome modification based on HDR is advantageous compared to NHEJ as it allows for the precise introduction of any desired genetic engineering, such as point mutations and eliminations.

Since the efficiency of HDR is substantially lower than NHEJ, alternative strategies have been developed for the targeted insertion of sequences by NHEJ using engineered nucleases, where the exogenous DNA fragment is directly linked to DSBs [150,155].

The Cas9/RecA system could potentially act as a therapeutic approach to correct genetic mutations for RP and many other inherited diseases. The incessant development of this technology to characterize and improve the safety and efficiency of genetic editing will help to fulfill its promise in the treatment of genetic diseases [154] (Table 1).

In the next part of the review, we focus on IRDs that have already been subjects of clinical trials. They will be classified on the basis of histogenesis.

## 8. Retinal Pigment Epithelium Disorders

### 8.1. Leber Congenital Amaurosis (LCA)

LCA is a severe congenital retinal dystrophy and, currently, the only treatable IRD due to the success of gene therapy. Children present early-onset marked vision loss, nystagmus, concentrically reduced visual field, and night blindness. Reduced or absent ERG responses and characteristic funduscopic signs are hallmarks of the condition in the first years of life. Progression is very fast, and patients rapidly experience blindness during early adulthood. To date, 26 different genes have been reported to be associated with LCA (RetNet. Available at: https://sph.uth.edu/retnet/). Among these genes, the *RPE65* gene, expressed in RPE cells, encodes a 65 kDa specific protein that catalyzes the conversion of all-trans retinyl esters to 11-cis retinal [156]. Without 11-cis retinal, opsins cannot capture light and convert it to electrical impulses. Loss of RPE65 disrupts the visual cycle, causing accumulation of retinyl esters in lipid droplets and an increase in lipofuscin granules in the RPE cells. The result is progressive retinal degeneration and loss of vision. About 4–16% of patients with LCA subtype 2 (LCA2) are found to have mutations in *RPE65*. Preclinical models of *RPE65*-related degeneration have been produced in order to move onto clinical trials [93] and, among them, canine [157], murine (rd12) [158], and the genetically built *Rpe65*^−/^^−^ knockout mouse [159] mimic the retinal degeneration seen in the disease. Indeed, *RPE65*-LCA clinical trials were initiated in 2007 by groups from the United Kingdom and the United States [83,160,161] following the encouraging results of animal studies. Successful gene therapy, such as through voretigene neparvovec under the tradename of Luxturna [162], is nowadays available for the cure of *RPE65* retinal dystrophy, the only treatable IRD so far. The long-term sustained effects on safety and efficacy of AAV2-*RPE65* gene delivery in biallelic *RPE65*-mediated LCA were assessed over a period of three years with the initial improvements in visual function peaking at 6–12 months after injection [163,164]. Spark Therapeutics continues to conduct clinical trials to determine the long-term effects of Luxturna (NCT03602820, NCT01208389, NCT00516477).

LCA subtype 10 (LCA10) is the most frequent form of LCA, affecting one-third of patients, and is caused by mutations in the *CEP290* gene; the most common mutation is the IVS26 c.2991+1655 A>G mutation. A CRISPR system was used to deliver two gRNAs into human fibroblasts from LCA10 patients with homozygous IVS26 mutations with consequent increases in wild-type *CEP290* expression [165]. Edited gRNAs and SaCas9 packaged into AAV5 vectors were later successfully delivered into transgenic mice containing the human ISV26 mutation via subretinal injection [46,47]. Once QR-110 oligonucleotide, designed for correction of ISV26 splicing defect, was shown to restore mRNA and protein expression levels in LCA10 fibroblasts [48], intravitreal injection of QR-110 was performed and well tolerated by nonhuman primates. A phase I/II trial to study the safety and tolerability of intravitreally administered QR-110 (NCT03140969) resulted in no serious adverse events and vision improvement at 3 months, with one exceptional responder from light perception to 20/400 of visual acuity [166]. A phase II/III trial has been started to evaluate the efficacy, safety, tolerability, and systemic exposure of QR-110 administered via intravitreal injection in subjects with LCA due to the *CEP290* p.Cys998X mutation after 24 months of treatment (NCT03913143).

### 8.2. Choroideremia (CHM)

This degenerative X-linked disease produces mainly degeneration of RPE, choriocapillaris, and photoreceptors in males [167]. Patients experience progressively reduced night vision and peripheral vision loss during adolescence and various degree of visual impairment, up to blindness, in the late stages of life. Female carriers may also show variable degrees of disease [168]. Mutations in the *CHM* gene, which encodes the Rab escort protein 1 (REP1) involved in post-translational lipid modification of Rab GTPases, are associated with this IRD, and are lacking in protein trafficking [169]. The AAV2-CHM virus was delivered in normal sighted mice and zebrafish, and the *CHM* gene was also induced in pluripotent stem cells (iPSCs) from patients with choroideremia with no evident toxicity [49,50], thus encouraging in vivo subretinal injection of the native AAV2-h*CHM* gene, which was reported to be safe and efficacious (NCT01461213 and NCT02341807) [170] for up to 3.5 years after treatment, despite progressive degeneration in the control eyes [171,172]. These encouraging results in preclinical studies led to a clinical trial of vectorized (AAV-REP1) wild-type *CHM* injected into the subretinal space. A phase III clinical trial comparing high and low titers of recombinant AAV2.REP1 to choroideremia patients is now ongoing (NCT03496012).

## 9. Photoreceptor Disorders

### 9.1. Retinitis Pigmentosa (RP)

RP is the most common IRD, occurring approximately in 1 out of 3500 births. It is characterized by progressive loss of photoreceptor function and structural integrity in patients who complain of night blindness and constriction of the visual field [173]. Almost 90 genes have been associated with RP (RetNet. Available at: https://sph.uth.edu/retnet/). Among these, mutations in the cGMP phosphodiesterase (*PDE*) gene can be found in autosomal recessive inheritance patterns. Intraocular administration of the normal *PDE6B* gene in a mouse model preserved retinal functions and prevented photoreceptor degeneration [52]. A phase I/II trial for subretinal administration of AAV2/5-hPDE6B has recently started (NCT03328130). This vector intends to supply to the target cells the *PDE6B* therapeutic gene and is now open to enrollment.

X-linked RP accounts for 10–20% of forms of this degeneration, and mutations in the RPGTPase regulator gene (*RPGR*) can be found in about 70% of XLRP patients. *RPGR* gene canine models (XLPRA1 and XLPRA2) have been treated with subretinal AAV2/5 full-length human RPGRex1-ORF15, with beneficial rescue of photoreceptor dysfunction to halt retinal degeneration [53]. Trials are ongoing to fully characterize clinical conditions, testing variability and rates of progression in subjects with RPGR-ORF15 mutations (NCT03314207) to evaluate the safety and efficacy of administration of AA2/5-hRKp.RPGR in XLRP patients (NCT03252847) and to test dose-escalation subretinal delivery of AAV8-RPGR in male subjects with genetically confirmed XLRP (NCT03116113). In addition, Applied Genetic Technologies corporation has announced a trial with the rAAv2tYF-GRK1-RP-GR vector.

Another target for RP gene therapy is the RPE-expressed “human receptor tyrosine kinase MER” (*MERTK*) gene. The inheritance pattern of mutations in this gene is autosomal recessive and almost 3% of ARRP cases are due to *MERTK* gene variants producing fast cone/rod degeneration [174,175]. Subretinal administration of AAV2, an AVV8-MERTK construct in an RGC rat model, prevented photoreceptor degeneration [55,104], and a phase I clinical trial in humans (NCT01482195) was initiated in Saudi Arabia on the basis of consistent experimental results by using subretinal administration of rAAV2-VMD2-hMERTK [176].

### 9.2. Usher Syndrome

Usher syndrome type 1 is an early-onset, syndromic form of RP, associated with bilateral neurosensorial deafness. Clinical features are profound hearing loss at birth, early-onset RP, and possibly decreased vestibular function [177]. Among the identified disease-causing genes [178], the *MYO7A* gene has been studied in gene therapy trials. Since it is a large gene, and therefore difficult to package into the AAV vector, UshStat, an EIAV lentiviral vector carrying the wild-type *MYO7A* gene (EIAV-CMV-MYO7A), was developed and injected in the *shaker1* mouse model for Usher syndrome type 1B [56]. In ongoing phase I/II Sanofi-sponsored trials, UshStat (SAR421869) is being injected subretinally into either Usher syndrome or RP patients (NCT02065011, NCT01505062) to test tolerability and safety.

### 9.3. Stargardt Maculopathy

Stargardt disease is the most common hereditary macular dystrophy caused by recessive mutations in the *ABCA4* gene [179]. Prototypical features of this central retinal degeneration are photofobia, early onset bilateral central vision loss, and progressive loss of outer retinal function and structure up to macular atrophy [180]. The *ABCA4* gene, the so-called “master gene” of the outer retina, encodes a membrane transporter protein localized in the outer segment of rod and cone photoreceptors. It is too large to be linked to an AAV vector [19]. Preclinical trials have used lentiviral gene therapy in mouse models to deliver the corrected *ABCA4* gene, resulting in reduced lipofuscin accumulation [57]. To evaluate the safety and efficacy of subretinal lentivirus-mediated administration of the *ABCA4* gene (SAR422459) to the human retina, a Sanofi-sponsored phase I/II clinical trial has been initiated (NCT01367444) [181]. Another phase I/II trial has evaluated the tolerability and safety of subretinal transplantation of hESC-derived RPE cells (MA09-hRPE) (NCT01345006). No immune response-adverse effects were observed and three out of seven enrolled patients experienced increased visual acuity during the 12 month trial period [182].

### 9.4. Achromatopsia

Achromatopsia is a rare IRD with very few visible signs of cone degeneration at fundoscopy. It is an autosomal recessive disorder characterized by photofobia, nystagmus, color blindness, and very low visual acuity [183]. Almost 50% of the encoding mutations belong to the causative *CNGB3* gene. Several animal studies have shown that a gene replacement approach can ameliorate photoreceptor function, and rodent, canine, and sheep models have been used to study mutations in the *CNGA3* gene [58]. The common opinion to date is that gene therapy for achromatopsia will need to be applied early in childhood to be effective. Several phase I/II trials in the United States (NCT02599922) and UK (NCT03001310) have been designed to test safety and efficacy of a subretinal injection rAAV vector delivering *hCNGB3*, and parallel studies have been conducted on the *CNGA3* gene in Germany (NCT02610582), the United States, and Israel (NCT02935517).

## 10. Inner Retina Disorders

### X-Linked Retinoschisis

X-linked retinoschisis (XLRS) is the most prevalent juvenile vitreoretinal degeneration in males and is caused by mutations in an extracellular adhesion protein, retinoschisin (RS1), normally expressed in the retina by photoreceptors and bipolar, amacrine, and ganglion cells [184]. Mutations in the *RS1* gene can produce a phenotype consisting of bilateral macular or peripheral retinal splitting of retinal layers, called schisis, as well as vitreous veils, increased incidence of retinal detachment, vitreous hemorrhages, and progressive retinal dystrophy up to macular atrophy, causing progressive visual loss [185]. Preclinical XLRS studies have shown that intravitreal injection of AAV8-scRS/IRBPhRS vector administration [59] as well as subretinal injection of the AAV5-mOPs-RS1 [60,186] produced significant improvement in retinal structure and function in the retinoschisin knockout (*Rs1-KO*) mouse, and good tolerability in rabbits [61]. Two phase I/II trials by intravitreal *RS1* delivery have been initiated with different constructs, AAV8-scRS/IRBPhRS in adults (NCT02317887), which have already reported encouraging initial findings [187], and rAAV2tYF-CB-hRS1 in children up to 6 years old (NCT02416622).

## 11. Conclusions

IRDs are a large and heterogeneous group of neurodegenerative disorders with great negative impacts with respect to patient sight and autonomy. Mutations of over 270 genes have been related to the pathogenesis of bilateral retinal dystrophies. Therapeutic avenues are being explored, as the majority of IRDs are considered orphan diseases. Several clinical studies, including gene therapy trials, are promising to regain visual function in absence of ocular and systemic side effects. The safety and efficacy of these treatments offer real hope in achieving a cure for IRDs patients. Since several pathways may be involved in the progressive loss of photoreceptors despite treatment, personalized and combined therapeutic strategies could be the answer to definitively halting degeneration in these highly heterogeneous genetic disorders.

## Figures and Tables

**Table 1:** Main features of gene therapy approaches discussed in this review.

Viral Mediated Therapy (Adenovirus, Adeno-Associated Virus, Lentivirus)				
	Gene Replacement	Gene Silencing	Therapeutic Oligo Nucleotides	Genome editing the CRISPR-Cas9 system
Integration Into Target Cell Genome	No Yes (LVV) ^a^	No Yes (LVV) ^a^	No Yes (LVV) ^a^	No Yes (LVV) ^a^
Lifetime Transgene Expression	Transient	-	-	Prolonged
Immunogenic Response	Yes	Yes	Yes	Yes
Mis-Insertion Risk	Yes (LVV) ^a^	Yes (LVV) ^a^	No	No
Development Costs	High	High	High	High
LoF ^c^ Mutation	Yes	No	Yes (AON) ^b^	Yes
GoF ^d^ Mutation	No	Yes	Yes	Yes
**Nanoparticles Mediated Therapy** **(Naked-DNA, Liposome-DNA, Metal-DNA)**				
	Gene Replacement	Gene Silencing	Therapeutic Oligonucleotides	Genome editing the CRISPR-Cas9 system
Integration Into Target Cell Genome	No	No	No	No
Immunogenic Response	None	None	None	None
Lifetime Transgene Expression	Transient	-	-	Prolonged
Mis-Insertion Risk	No	No	No	No
Development Costs	Low	Low	Low	Low
LoF ^c^ Mutation	Yes	No	Yes (AON) ^b^	Yes
GoF ^d^ Mutation	No	Yes	Yes	Yes

^a^ LVV = Lentiviral vector; ^b^ AON = Antisense oligonucleotides; ^c^ LoF= Loss of function; ^d^ GoF= Gain of function.

**Table 2:** Major preclinical models for treatment of inherited retinal dystrophies.

Retinal Dystrophy	Gene	Animal Model	Construct
Leber congenital amaurosis	*RPE65*	*rd12* mouse, *Rpe65*^−/−^ mouse *rd12* mouse *rd12* mouse	AAV2.RPE65 [43] scAAV5-smCBA-hRPE65 [44] rAAV5-CBA-hRPE65 [45]
	*CEP290*	mice containing the human ISV26 Wild-type mouse and rabbit, non-human primates HuCEP290 knock-in mice	gRNAs and SaCas9 packaged into AAV5 vectors [46,47] QR-110 [48] Edit-101 [47]
Choriodermia	*CHM*	normal-sighted mice chm^ru848^ zebrafish chm^ru848^ zebrafish	rAAV-CHM virus [49] PTC124 and PTC-414 [50] PTC124 and PTC-414 [51]
Retinitis pigmentosa	*PDE6B*	*rd10* mouse	pEPito-hCMVeGFP [52]
	*RPGR*	Canine models XLPRA1/2	AAV2/5-hIRBP-hRPGR, AAV2/5-hGRK1-hRPGR [53]
	*MERTK*	Royal College of Surgeons rat Royal College of Surgeons rat	rAAV-MERTK [54] AAV8-Y733F-MERTK [55]
Usher Syndrome	*MYO7A*	*shaker1* mouse	EIAV-CMV-MYO7A [56]
Stargardt maculopathy	*ABCA4*	*Abca4*^−/−^ mouse	EIAV-CMV-ABCA4 [57]
Achromatopsia	*CNGA3*	*Cnga3*^–^^/–^ mouse	rAAV.CBA.CNGA3 [58]
X-linked retinoschisis	*RS1*	*Rs1-KO* mouse New Zealand white rabbits, *Rs1-KO* mouse	AAV8-scRS/IRBPhRS [59] AAV5-mOP-RS1 [60] AAV8-scRS/IRBPhRS [61]
